# 3-Isopropyl-1-{2-[(1-methyl-1*H*-tetra­zol-5-yl)sulfan­yl]acet­yl}-2,6-di­phenyl­piperidin-4-one hemihydrate

**DOI:** 10.1107/S1600536813026500

**Published:** 2013-10-02

**Authors:** S. Ganesan, P. Sugumar, S. Ananthan, M. N. Ponnuswamy

**Affiliations:** a Research Development Centre, Orchid Chemicals and Pharmaceuticals Ltd, Sozhinganallur, Chennai 600 119, India; b Department of Chemistry, Presidency College (Autonomous), Chennai 600 005, India; c Centre of Advanced Study in Crystallography and Biophysics, University of Madras, Guindy Campus, Chennai 600 025, India

## Abstract

In the title compound, C_24_H_27_N_5_O_2_S·0.5H_2_O, the piperidine ring adopts a distorted boat conformation. The phenyl rings subtend dihedral angles of 69.7 (1) and 88.7 (1)° with the best plane through the piperidine moiety. In the crystal, symmetry-related mol­ecules are linked through a network of C—H⋯O and C—H⋯N inter­actions, the former connecting them into zigzag chains along the *c*-axis direction and the latter forming an *R* ^2^_2_(4)motif. The dimer formation (C—H⋯N) and the repetition of symmetry-related molecules (C—H⋯O) along the *b*-axis direction stabilize the packing mode. The water mol­ecule is located on a twofold rotation axis.

## Related literature

For the biological activity of piperidine derivatives, see: Aridoss *et al.* (2009 ▶). For puckering parameters, see: Cremer & Pople (1975 ▶). For asymmetry parameters, see: Nardelli (1983 ▶). For hydrogen-bond motifs, see: Bernstein *et al.* (1995 ▶).
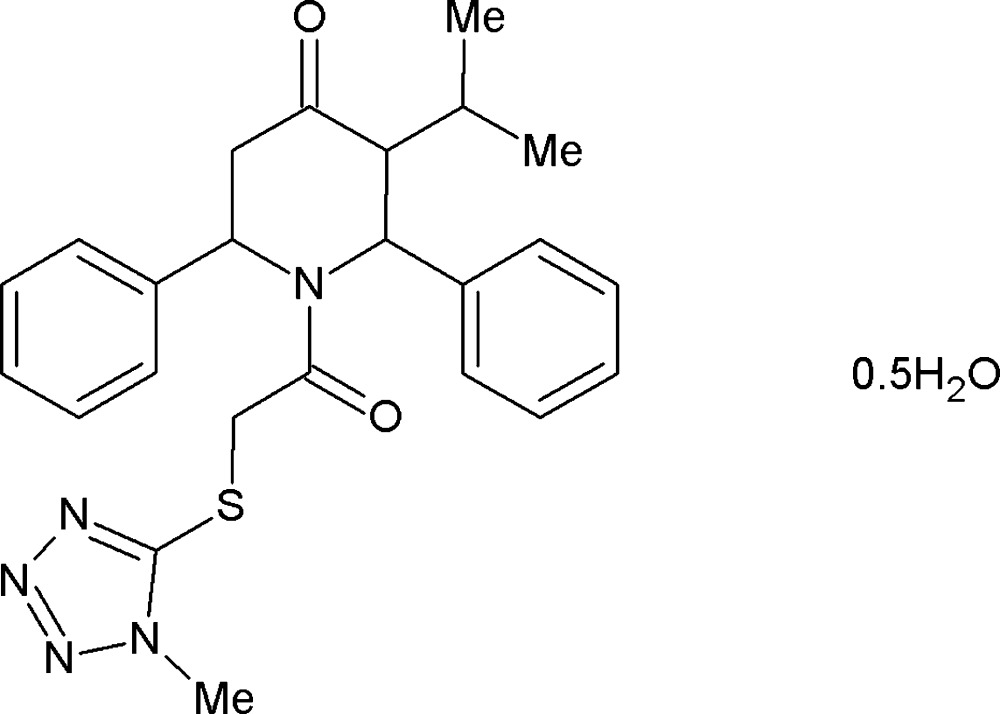


## Experimental

### 

#### Crystal data

C_24_H_27_N_5_O_2_S·0.5H_2_O
*M* *_r_* = 458.57
Monoclinic, 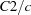

*a* = 28.7522 (9) Å
*b* = 11.1809 (4) Å
*c* = 16.5584 (5) Å
β = 115.303 (2)°
*V* = 4812.4 (3) Å^3^
*Z* = 8
Mo *K*α radiation
μ = 0.17 mm^−1^
*T* = 293 K
0.22 × 0.19 × 0.17 mm

#### Data collection

Bruker SMART APEXII CCD diffractometer
Absorption correction: multi-scan (*SADABS*; Bruker, 2008 ▶) *T*_min_ = 0.964, *T*_max_ = 0.972
22058 measured reflections
6013 independent reflections
4157 reflections with *I* > 2σ(*I*)
*R*_int_ = 0.026

#### Refinement

*R*[*F* ^2^ > 2σ(*F* ^2^)] = 0.046
*wR*(*F* ^2^) = 0.146
*S* = 1.04
6013 reflections
297 parameters
H-atom parameters constrained
Δρ_max_ = 0.42 e Å^−3^
Δρ_min_ = −0.25 e Å^−3^

### 

Data collection: *APEX2* (Bruker, 2008 ▶); cell refinement: *SAINT* (Bruker, 2008 ▶); data reduction: *SAINT*; program(s) used to solve structure: *SHELXS97* (Sheldrick, 2008 ▶); program(s) used to refine structure: *SHELXL97* (Sheldrick, 2008 ▶); molecular graphics: *ORTEP-3 for Windows* (Farrugia, 2012 ▶); software used to prepare material for publication: *SHELXL97* and *PLATON* (Spek, 2009 ▶).

## Supplementary Material

Crystal structure: contains datablock(s) global, I. DOI: 10.1107/S1600536813026500/bt6930sup1.cif

Structure factors: contains datablock(s) I. DOI: 10.1107/S1600536813026500/bt6930Isup2.hkl

Supplementary material file. DOI: 10.1107/S1600536813026500/bt6930Isup3.cml

Additional supplementary materials:  crystallographic information; 3D view; checkCIF report

## Figures and Tables

**Table 1:** Hydrogen-bond geometry (Å, °)

*D*—H⋯*A*	*D*—H	H⋯*A*	*D*⋯*A*	*D*—H⋯*A*
C25—H25*C*⋯O1^i^	0.96	2.47	3.406 (3)	166
C18—H18⋯O1^ii^	0.93	2.54	3.312 (2)	140
C25—H25*B*⋯N4^iii^	0.96	2.54	3.472 (3)	165

Symmetry codes: (i) 
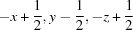
; (ii) 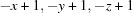; (iii) 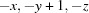.

**Fig. 1.**
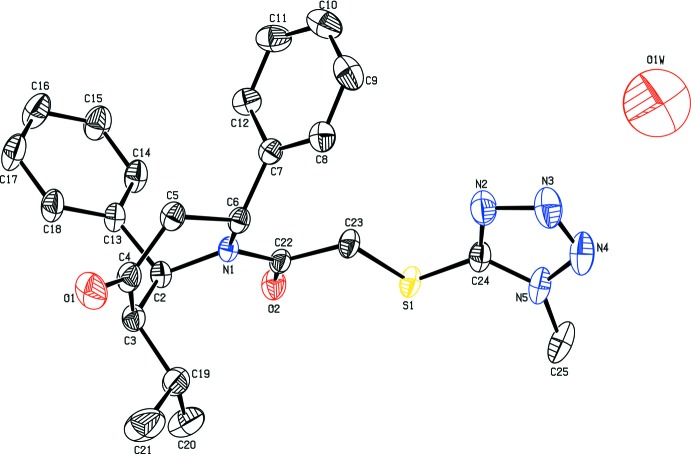
The molecular structure of the title compound, showing the atomic numbering and displacement ellipsoids drawn at 30% probability level.

**Fig. 2.**
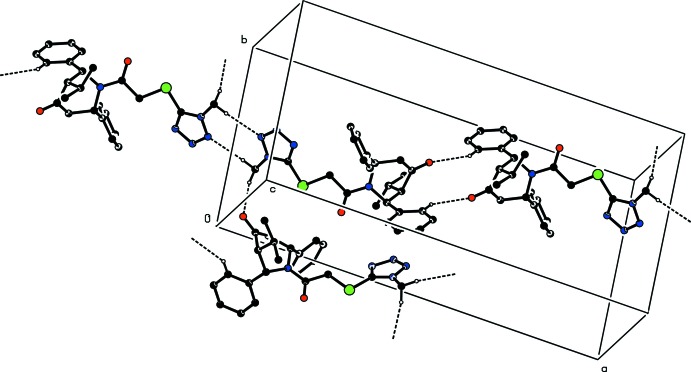
The crystal packing of the molecules viewed down c axis.

## supplementary crystallographic information

### 1. Comment

In a way to find piperidin-4-one based lead drug molecules for the antimicrobial therapy, various piperidin-4-ones were prepared by condensing *N*-chloroacetyl-2,6-diphenylpiperidin-4-one with 5-mercapto-(1-methyltetrazole) (Aridoss *et al.*, 2009). 5-Mercapto-(1-methyl tetrazole) is the active part of a number of cephalosporanic drugs like Cefamandole, Cefoperazone, Cefmetazole sodium & Cefotetan and responsible for its activity. The present investigation was undertaken to establish the structure and conformation of the title compound by X-ray crystallographic methods.

The *ORTEP* plot of the molecule is shown in Fig. 1. The piperidine ring adopts a distorted boat conformation with the puckering parameters (Cremer & Pople, 1975) and the asymmetry parameters (Nardelli, 1983): q_2_=0.662 (2) Å, q_3_ = 0.079 (2) Å, φ_2_ = 76.86 (2)° and Δ_s_(N1 & C4)= 74.11 (2)°.

The methyltetrazol ring is planar with the maximum deviation -0.003 (2) Å for N4 atom. The endocyclic bond lengths of N2–N3=1.362 (4)°, N3–N4= 1.278 (4) Å & N4–N5 = 1.348 (3) Å, clearly indicate that they are alternate single and double bonds.

The carbonyl group is almost *anti-periplanar* to C2 and C6, [C2—C3—C4—O1 = 155.9 (2)°; C6—C5—C4—O1= 151.1 (2)°]. The dihedral angles between the best plane through the piperidine ring and the phenyl rings are 69.7 (1)° & 88.7 (1)°. The two phenyl rings are oriented to each other with a dihedral angle of 70.5 (1)°.

Symmetry related molecules are linked through a network of intermolecular C—H···O and C—H···N interactions. C18–H18···O1 and C25–H25B···O1 connect the molecules to zigzag chains. Another motif *R*^2^_2_(4), involving the weak interaction C25—H25A···N4 is shown in Fig. 2 (Bernstein *et al.*, 1995).

### 2. Experimental

To anhydrous DMF(10 ml), *N*-Chloroacetyl-3-isopropyl-2,6-diphenylpiperidin-4-one (1 mole), 5-Mercapto-1-methyltetrazole(1 mole) followed by potassium carbonate (1.5 mole) was added and stirred for 1 hr at room temperature. The reaction mass was heated to 60°C and stirred and monitored using TLC. After completion of reaction, the reaction mass was quenched into water and the product was extracted with dichloromethane. The dichloromethane layer distilled completely and to the residue methanol was added and kept in overnight. The solid obtained was filtered and dried at 60° C under vacuum. Single crystals were obtained by re-crystallization using ethanol.

### 3. Refinement

H atoms were positioned geometrically (C–H = 0.93–0.98 Å) and allowed to ride on their parent atoms, with *U*_iso_(H) =1.5*U*_eq_(C) for methyl H atoms and 1.2*U*_eq_(C) for all other H atoms. The ADP value for the water molecules is rather high. Since the H atoms of the solvent water molecule could not be located, they were not included in the refinement.

### Crystal data

**Table:**

C_24_H_27_N_5_O_2_S·0.5H_2_O	*F*(000) = 1944
*M**_r_* = 458.57	*D*_x_ = 1.266 Mg m^−^^3^
Monoclinic, *C*2/*c*	Mo *K*α radiation, λ = 0.71073 Å
Hall symbol: -C 2yc	Cell parameters from 4157 reflections
*a* = 28.7522 (9) Å	θ = 2.5–28.5°
*b* = 11.1809 (4) Å	µ = 0.17 mm^−^^1^
*c* = 16.5584 (5) Å	*T* = 293 K
β = 115.303 (2)°	Block, white crystalline
*V* = 4812.4 (3) Å^3^	0.22 × 0.19 × 0.17 mm
*Z* = 8	

### Data collection

**Table:**

Bruker SMART APEXII CCD diffractometer	6013 independent reflections
Radiation source: fine-focus sealed tube	4157 reflections with *I* > 2σ(*I*)
Graphite monochromator	*R*_int_ = 0.026
ω and φ scans	θ_max_ = 28.5°, θ_min_ = 2.5°
Absorption correction: multi-scan (*SADABS*; Bruker, 2008)	*h* = −38→37
*T*_min_ = 0.964, *T*_max_ = 0.972	*k* = −14→14
22058 measured reflections	*l* = −22→22

### Refinement

**Table:**

Refinement on *F*^2^	Primary atom site location: structure-invariant direct methods
Least-squares matrix: full	Secondary atom site location: difference Fourier map
*R*[*F*^2^ > 2σ(*F*^2^)] = 0.046	Hydrogen site location: inferred from neighbouring sites
*wR*(*F*^2^) = 0.146	H-atom parameters constrained
*S* = 1.04	*w* = 1/[σ^2^(*F*_o_^2^) + (0.0657*P*)^2^ + 3.1292*P*] where *P* = (*F*_o_^2^ + 2*F*_c_^2^)/3
6013 reflections	(Δ/σ)_max_ < 0.001
297 parameters	Δρ_max_ = 0.42 e Å^−^^3^
0 restraints	Δρ_min_ = −0.25 e Å^−^^3^

### Special details

**Table:**

Geometry. All e.s.d.'s (except the e.s.d. in the dihedral angle between two l.s. planes) are estimated using the full covariance matrix. The cell e.s.d.'s are taken into account individually in the estimation of e.s.d.'s in distances, angles and torsion angles; correlations between e.s.d.'s in cell parameters are only used when they are defined by crystal symmetry. An approximate (isotropic) treatment of cell e.s.d.'s is used for estimating e.s.d.'s involving l.s. planes.
Refinement. Refinement of *F*^2^ against ALL reflections. The weighted *R*-factor *wR* and goodness of fit *S* are based on *F*^2^, conventional *R*-factors *R* are based on *F*, with *F* set to zero for negative *F*^2^. The threshold expression of *F*^2^ > σ(*F*^2^) is used only for calculating *R*-factors(gt) *etc*. and is not relevant to the choice of reflections for refinement. *R*-factors based on *F*^2^ are statistically about twice as large as those based on *F*, and *R*- factors based on ALL data will be even larger.

### Fractional atomic coordinates and isotropic or equivalent isotropic displacement parameters (Å^2^)

**Table:**

	*x*	*y*	*z*	*U*_iso_*/*U*_eq_	
C2	0.36794 (6)	0.31060 (16)	0.37251 (12)	0.0412 (4)	
H2	0.3603	0.2457	0.3289	0.049*	
C3	0.40052 (7)	0.40262 (18)	0.35071 (12)	0.0492 (4)	
H3	0.4339	0.3659	0.3635	0.059*	
C4	0.41000 (7)	0.51029 (18)	0.41040 (13)	0.0519 (5)	
C5	0.37256 (7)	0.52979 (16)	0.45051 (12)	0.0465 (4)	
H5A	0.3715	0.6147	0.4617	0.056*	
H5B	0.3856	0.4894	0.5078	0.056*	
C6	0.31739 (6)	0.48676 (14)	0.39456 (11)	0.0388 (4)	
H6	0.3012	0.5417	0.3441	0.047*	
C7	0.28781 (6)	0.49375 (15)	0.45121 (11)	0.0402 (4)	
C8	0.25431 (7)	0.58770 (17)	0.43993 (14)	0.0519 (5)	
H8	0.2485	0.6437	0.3951	0.062*	
C9	0.22951 (9)	0.5988 (2)	0.49479 (18)	0.0661 (6)	
H9	0.2071	0.6625	0.4868	0.079*	
C10	0.23757 (9)	0.5170 (2)	0.56082 (17)	0.0682 (6)	
H10	0.2211	0.5253	0.5981	0.082*	
C11	0.27031 (9)	0.4222 (2)	0.57168 (15)	0.0638 (6)	
H11	0.2755	0.3657	0.6159	0.077*	
C12	0.29535 (8)	0.41052 (17)	0.51762 (13)	0.0508 (4)	
H12	0.3175	0.3464	0.5257	0.061*	
C13	0.39463 (6)	0.25381 (16)	0.46536 (12)	0.0439 (4)	
C14	0.36763 (7)	0.16991 (17)	0.48954 (14)	0.0525 (5)	
H14	0.3345	0.1489	0.4490	0.063*	
C15	0.38885 (9)	0.1164 (2)	0.57293 (15)	0.0640 (6)	
H15	0.3698	0.0611	0.5883	0.077*	
C16	0.43792 (9)	0.1451 (2)	0.63289 (16)	0.0708 (6)	
H16	0.4522	0.1097	0.6891	0.085*	
C17	0.46572 (8)	0.2261 (2)	0.60943 (16)	0.0712 (7)	
H17	0.4991	0.2450	0.6498	0.085*	
C18	0.44445 (7)	0.28039 (19)	0.52588 (14)	0.0570 (5)	
H18	0.4638	0.3349	0.5106	0.068*	
C19	0.37600 (8)	0.4421 (2)	0.25143 (14)	0.0598 (5)	
H19	0.3441	0.4849	0.2402	0.072*	
C20	0.36228 (12)	0.3358 (3)	0.18807 (16)	0.0836 (8)	
H20A	0.3481	0.3639	0.1275	0.125*	
H20B	0.3374	0.2867	0.1968	0.125*	
H20C	0.3927	0.2896	0.2000	0.125*	
C21	0.41134 (12)	0.5283 (3)	0.2321 (2)	0.1060 (11)	
H21A	0.4432	0.4888	0.2434	0.159*	
H21B	0.4181	0.5971	0.2701	0.159*	
H21C	0.3950	0.5530	0.1707	0.159*	
C22	0.27371 (6)	0.31101 (15)	0.30215 (11)	0.0386 (4)	
C23	0.22425 (6)	0.37998 (17)	0.27937 (12)	0.0456 (4)	
H23A	0.2283	0.4620	0.2643	0.055*	
H23B	0.2159	0.3808	0.3303	0.055*	
C24	0.12585 (7)	0.41117 (17)	0.17409 (13)	0.0499 (4)	
C25	0.06530 (10)	0.3638 (2)	0.01369 (17)	0.0818 (8)	
H25A	0.0921	0.3667	−0.0062	0.123*	
H25B	0.0352	0.4030	−0.0292	0.123*	
H25C	0.0573	0.2819	0.0199	0.123*	
N1	0.31787 (5)	0.36569 (12)	0.35824 (9)	0.0368 (3)	
N2	0.12550 (7)	0.48754 (19)	0.23410 (14)	0.0695 (5)	
N3	0.08016 (8)	0.5478 (2)	0.19371 (18)	0.0833 (6)	
N4	0.05421 (7)	0.51015 (18)	0.11397 (18)	0.0799 (6)	
N5	0.08241 (6)	0.42381 (15)	0.09937 (12)	0.0600 (5)	
O1	0.44618 (6)	0.57684 (16)	0.42809 (13)	0.0792 (5)	
O2	0.27217 (5)	0.21064 (12)	0.27106 (9)	0.0508 (3)	
S1	0.173492 (17)	0.30767 (4)	0.18556 (3)	0.04999 (15)	
O1W	0.0000	0.7061 (7)	0.2500	0.373 (7)	
H1W	0.0172	0.6692	0.2298	0.560*	

### Atomic displacement parameters (Å^2^)

**Table:**

	*U*^11^	*U*^22^	*U*^33^	*U*^12^	*U*^13^	*U*^23^
C2	0.0302 (8)	0.0449 (9)	0.0444 (9)	−0.0009 (7)	0.0121 (7)	−0.0003 (7)
C3	0.0349 (9)	0.0609 (12)	0.0488 (10)	−0.0035 (8)	0.0152 (8)	0.0055 (8)
C4	0.0369 (9)	0.0564 (11)	0.0538 (11)	−0.0119 (8)	0.0110 (8)	0.0054 (8)
C5	0.0403 (9)	0.0427 (9)	0.0481 (9)	−0.0116 (7)	0.0109 (8)	−0.0033 (7)
C6	0.0345 (8)	0.0355 (8)	0.0397 (8)	−0.0034 (6)	0.0093 (7)	0.0020 (6)
C7	0.0379 (8)	0.0378 (9)	0.0405 (8)	−0.0080 (7)	0.0125 (7)	−0.0056 (6)
C8	0.0477 (10)	0.0447 (10)	0.0582 (11)	−0.0021 (8)	0.0178 (9)	−0.0059 (8)
C9	0.0559 (12)	0.0567 (13)	0.0906 (16)	−0.0078 (10)	0.0360 (12)	−0.0249 (12)
C10	0.0721 (14)	0.0690 (14)	0.0807 (15)	−0.0302 (12)	0.0491 (13)	−0.0313 (12)
C11	0.0796 (15)	0.0617 (13)	0.0586 (12)	−0.0225 (11)	0.0377 (12)	−0.0074 (10)
C12	0.0568 (11)	0.0444 (10)	0.0514 (10)	−0.0058 (8)	0.0234 (9)	−0.0009 (8)
C13	0.0315 (8)	0.0431 (9)	0.0488 (9)	0.0015 (7)	0.0091 (7)	0.0029 (7)
C14	0.0369 (9)	0.0475 (10)	0.0594 (11)	−0.0039 (8)	0.0076 (9)	0.0079 (8)
C15	0.0550 (12)	0.0554 (12)	0.0684 (13)	−0.0029 (10)	0.0138 (11)	0.0173 (10)
C16	0.0609 (13)	0.0701 (14)	0.0577 (12)	0.0018 (11)	0.0028 (11)	0.0188 (11)
C17	0.0433 (11)	0.0793 (15)	0.0615 (13)	−0.0053 (11)	−0.0057 (10)	0.0118 (11)
C18	0.0340 (9)	0.0624 (12)	0.0614 (12)	−0.0061 (8)	0.0077 (9)	0.0094 (10)
C19	0.0508 (11)	0.0727 (14)	0.0510 (11)	−0.0044 (10)	0.0170 (9)	0.0103 (10)
C20	0.096 (2)	0.0965 (19)	0.0498 (12)	−0.0058 (16)	0.0236 (13)	0.0015 (12)
C21	0.104 (2)	0.139 (3)	0.0702 (16)	−0.043 (2)	0.0331 (16)	0.0237 (17)
C22	0.0305 (8)	0.0454 (9)	0.0335 (7)	−0.0032 (7)	0.0076 (6)	0.0004 (7)
C23	0.0312 (8)	0.0503 (10)	0.0426 (9)	−0.0025 (7)	0.0036 (7)	−0.0051 (8)
C24	0.0332 (9)	0.0517 (10)	0.0541 (10)	−0.0021 (8)	0.0085 (8)	0.0071 (8)
C25	0.0601 (14)	0.0748 (16)	0.0668 (14)	0.0057 (12)	−0.0146 (12)	0.0038 (12)
N1	0.0279 (6)	0.0386 (7)	0.0373 (7)	−0.0025 (5)	0.0077 (5)	−0.0008 (5)
N2	0.0495 (10)	0.0756 (13)	0.0749 (12)	0.0097 (9)	0.0185 (9)	−0.0033 (10)
N3	0.0576 (12)	0.0761 (14)	0.1063 (17)	0.0158 (11)	0.0254 (12)	−0.0021 (13)
N4	0.0472 (10)	0.0623 (12)	0.1054 (17)	0.0130 (9)	0.0089 (11)	0.0096 (11)
N5	0.0376 (8)	0.0515 (9)	0.0681 (11)	0.0017 (7)	0.0009 (8)	0.0113 (8)
O1	0.0542 (9)	0.0838 (11)	0.0987 (12)	−0.0338 (8)	0.0320 (9)	−0.0138 (9)
O2	0.0399 (7)	0.0483 (7)	0.0540 (7)	−0.0035 (5)	0.0104 (6)	−0.0138 (6)
S1	0.0327 (2)	0.0534 (3)	0.0470 (3)	−0.00136 (19)	0.00093 (18)	−0.0049 (2)
O1W	0.275 (9)	0.202 (7)	0.68 (2)	0.000	0.242 (13)	0.000

### Geometric parameters (Å, º)

**Table:**

C2—N1	1.489 (2)	C16—C17	1.370 (3)
C2—C13	1.532 (2)	C16—H16	0.9300
C2—C3	1.535 (2)	C17—C18	1.390 (3)
C2—H2	0.9800	C17—H17	0.9300
C3—C4	1.507 (3)	C18—H18	0.9300
C3—C19	1.551 (3)	C19—C20	1.522 (3)
C3—H3	0.9800	C19—C21	1.530 (3)
C4—O1	1.209 (2)	C19—H19	0.9800
C4—C5	1.503 (3)	C20—H20A	0.9600
C5—C6	1.533 (2)	C20—H20B	0.9600
C5—H5A	0.9700	C20—H20C	0.9600
C5—H5B	0.9700	C21—H21A	0.9600
C6—N1	1.484 (2)	C21—H21B	0.9600
C6—C7	1.514 (2)	C21—H21C	0.9600
C6—H6	0.9800	C22—O2	1.228 (2)
C7—C8	1.383 (3)	C22—N1	1.357 (2)
C7—C12	1.385 (3)	C22—C23	1.517 (2)
C8—C9	1.379 (3)	C23—S1	1.8052 (17)
C8—H8	0.9300	C23—H23A	0.9700
C9—C10	1.367 (4)	C23—H23B	0.9700
C9—H9	0.9300	C24—N2	1.313 (3)
C10—C11	1.378 (3)	C24—N5	1.338 (2)
C10—H10	0.9300	C24—S1	1.741 (2)
C11—C12	1.374 (3)	C25—N5	1.452 (3)
C11—H11	0.9300	C25—H25A	0.9600
C12—H12	0.9300	C25—H25B	0.9600
C13—C14	1.382 (3)	C25—H25C	0.9600
C13—C18	1.385 (2)	N2—N3	1.362 (3)
C14—C15	1.384 (3)	N3—N4	1.279 (3)
C14—H14	0.9300	N4—N5	1.347 (3)
C15—C16	1.372 (3)	O1W—H1W	0.8163
C15—H15	0.9300		
			
N1—C2—C13	111.48 (14)	C17—C16—H16	120.2
N1—C2—C3	109.35 (14)	C15—C16—H16	120.2
C13—C2—C3	114.81 (14)	C16—C17—C18	120.61 (19)
N1—C2—H2	106.9	C16—C17—H17	119.7
C13—C2—H2	106.9	C18—C17—H17	119.7
C3—C2—H2	106.9	C13—C18—C17	120.36 (19)
C4—C3—C2	109.80 (15)	C13—C18—H18	119.8
C4—C3—C19	109.92 (17)	C17—C18—H18	119.8
C2—C3—C19	113.18 (15)	C20—C19—C21	110.3 (2)
C4—C3—H3	107.9	C20—C19—C3	111.99 (19)
C2—C3—H3	107.9	C21—C19—C3	111.05 (18)
C19—C3—H3	107.9	C20—C19—H19	107.8
O1—C4—C5	120.56 (19)	C21—C19—H19	107.8
O1—C4—C3	123.12 (19)	C3—C19—H19	107.8
C5—C4—C3	116.29 (15)	C19—C20—H20A	109.5
C4—C5—C6	116.04 (15)	C19—C20—H20B	109.5
C4—C5—H5A	108.3	H20A—C20—H20B	109.5
C6—C5—H5A	108.3	C19—C20—H20C	109.5
C4—C5—H5B	108.3	H20A—C20—H20C	109.5
C6—C5—H5B	108.3	H20B—C20—H20C	109.5
H5A—C5—H5B	107.4	C19—C21—H21A	109.5
N1—C6—C7	113.78 (13)	C19—C21—H21B	109.5
N1—C6—C5	110.17 (14)	H21A—C21—H21B	109.5
C7—C6—C5	108.64 (14)	C19—C21—H21C	109.5
N1—C6—H6	108.0	H21A—C21—H21C	109.5
C7—C6—H6	108.0	H21B—C21—H21C	109.5
C5—C6—H6	108.0	O2—C22—N1	123.69 (16)
C8—C7—C12	118.77 (18)	O2—C22—C23	119.90 (15)
C8—C7—C6	119.93 (16)	N1—C22—C23	116.41 (14)
C12—C7—C6	121.23 (16)	C22—C23—S1	108.28 (12)
C9—C8—C7	120.4 (2)	C22—C23—H23A	110.0
C9—C8—H8	119.8	S1—C23—H23A	110.0
C7—C8—H8	119.8	C22—C23—H23B	110.0
C10—C9—C8	120.5 (2)	S1—C23—H23B	110.0
C10—C9—H9	119.7	H23A—C23—H23B	108.4
C8—C9—H9	119.7	N2—C24—N5	108.97 (18)
C9—C10—C11	119.5 (2)	N2—C24—S1	127.57 (15)
C9—C10—H10	120.3	N5—C24—S1	123.46 (16)
C11—C10—H10	120.3	N5—C25—H25A	109.5
C12—C11—C10	120.5 (2)	N5—C25—H25B	109.5
C12—C11—H11	119.8	H25A—C25—H25B	109.5
C10—C11—H11	119.8	N5—C25—H25C	109.5
C11—C12—C7	120.4 (2)	H25A—C25—H25C	109.5
C11—C12—H12	119.8	H25B—C25—H25C	109.5
C7—C12—H12	119.8	C22—N1—C6	121.37 (13)
C14—C13—C18	118.14 (17)	C22—N1—C2	118.73 (14)
C14—C13—C2	118.02 (15)	C6—N1—C2	119.27 (13)
C18—C13—C2	123.84 (17)	C24—N2—N3	105.37 (19)
C13—C14—C15	121.32 (18)	N4—N3—N2	111.0 (2)
C13—C14—H14	119.3	N3—N4—N5	106.89 (18)
C15—C14—H14	119.3	C24—N5—N4	107.78 (19)
C16—C15—C14	120.0 (2)	C24—N5—C25	130.34 (19)
C16—C15—H15	120.0	N4—N5—C25	121.86 (18)
C14—C15—H15	120.0	C24—S1—C23	96.05 (9)
C17—C16—C15	119.6 (2)		
			
N1—C2—C3—C4	59.75 (18)	C2—C13—C18—C17	179.1 (2)
C13—C2—C3—C4	−66.4 (2)	C16—C17—C18—C13	0.4 (4)
N1—C2—C3—C19	−63.5 (2)	C4—C3—C19—C20	−176.12 (19)
C13—C2—C3—C19	170.36 (17)	C2—C3—C19—C20	−53.0 (2)
C2—C3—C4—O1	155.9 (2)	C4—C3—C19—C21	60.0 (3)
C19—C3—C4—O1	−79.0 (2)	C2—C3—C19—C21	−176.8 (2)
C2—C3—C4—C5	−22.2 (2)	O2—C22—C23—S1	−14.3 (2)
C19—C3—C4—C5	102.97 (19)	N1—C22—C23—S1	166.62 (12)
O1—C4—C5—C6	151.13 (19)	O2—C22—N1—C6	−179.37 (15)
C3—C4—C5—C6	−30.8 (2)	C23—C22—N1—C6	−0.3 (2)
C4—C5—C6—N1	45.1 (2)	O2—C22—N1—C2	9.8 (2)
C4—C5—C6—C7	170.36 (15)	C23—C22—N1—C2	−171.18 (14)
N1—C6—C7—C8	−135.30 (16)	C7—C6—N1—C22	61.59 (19)
C5—C6—C7—C8	101.58 (18)	C5—C6—N1—C22	−176.13 (14)
N1—C6—C7—C12	47.8 (2)	C7—C6—N1—C2	−127.61 (15)
C5—C6—C7—C12	−75.3 (2)	C5—C6—N1—C2	−5.3 (2)
C12—C7—C8—C9	0.9 (3)	C13—C2—N1—C22	−107.47 (17)
C6—C7—C8—C9	−176.10 (17)	C3—C2—N1—C22	124.49 (16)
C7—C8—C9—C10	−0.2 (3)	C13—C2—N1—C6	81.48 (17)
C8—C9—C10—C11	−0.7 (3)	C3—C2—N1—C6	−46.55 (19)
C9—C10—C11—C12	1.0 (3)	N5—C24—N2—N3	0.1 (2)
C10—C11—C12—C7	−0.3 (3)	S1—C24—N2—N3	−179.61 (17)
C8—C7—C12—C11	−0.7 (3)	C24—N2—N3—N4	0.3 (3)
C6—C7—C12—C11	176.30 (17)	N2—N3—N4—N5	−0.6 (3)
N1—C2—C13—C14	55.1 (2)	N2—C24—N5—N4	−0.4 (2)
C3—C2—C13—C14	−179.87 (17)	S1—C24—N5—N4	179.29 (15)
N1—C2—C13—C18	−125.88 (19)	N2—C24—N5—C25	177.7 (2)
C3—C2—C13—C18	−0.8 (3)	S1—C24—N5—C25	−2.5 (3)
C18—C13—C14—C15	2.1 (3)	N3—N4—N5—C24	0.6 (3)
C2—C13—C14—C15	−178.76 (19)	N3—N4—N5—C25	−177.7 (2)
C13—C14—C15—C16	−1.1 (4)	N2—C24—S1—C23	−19.8 (2)
C14—C15—C16—C17	−0.4 (4)	N5—C24—S1—C23	160.54 (17)
C15—C16—C17—C18	0.7 (4)	C22—C23—S1—C24	−176.64 (13)
C14—C13—C18—C17	−1.8 (3)		

### Hydrogen-bond geometry (Å, º)

**Table:**

*D*—H···*A*	*D*—H	H···*A*	*D*···*A*	*D*—H···*A*
C25—H25*C*···O1^i^	0.96	2.47	3.406 (3)	166
C18—H18···O1^ii^	0.93	2.54	3.312 (2)	140
C25—H25*B*···N4^iii^	0.96	2.54	3.472 (3)	165

Symmetry codes: (i) −*x*+1/2, *y*−1/2, −*z*+1/2; (ii) −*x*+1, −*y*+1, −*z*+1; (iii) −*x*, −*y*+1, −*z*.
